# Re‐adjudication of the Trial Evaluating Cardiovascular Outcomes with Sitagliptin (TECOS) with study‐level meta‐analysis of hospitalization for heart failure from cardiovascular outcomes trials with dipeptidyl peptidase‐4 (DPP‐4) inhibitors

**DOI:** 10.1002/clc.23844

**Published:** 2022-06-17

**Authors:** Benjamin M. Scirica, KyungAh Im, Sabina A. Murphy, Julia F. Kuder, Dolly A. Rodriguez, Renato D. Lopes, Jennifer B. Green, Christian T. Ruff, Marc S. Sabatine

**Affiliations:** ^1^ TIMI Study Group, Cardiovascular Medicine Division Brigham and Women's Hospital and Harvard Medical School Boston Massachusetts USA; ^2^ Duke Clinical Research Institute Durham North Carolina USA

**Keywords:** diabetes, dipeptidyl peptidase‐4 (DPP‐4) inhibitor, heart failure, meta‐analysis

## Abstract

**Background:**

Trial Evaluating Cardiovascular Outcomes with Sitagliptin (TECOS) assessed the cardiovascular (CV) safety of sitagliptin versus placebo on CV outcomes in patients with type 2 diabetes and CV disease and found sitagliptin noninferior to placebo. Subsequently, based on feedback from FDA, the Sponsor of the trial, Merck & Co., Inc., engaged a separate academic research organization, the TIMI Study Group, to re‐adjudicate a prespecified set of originally adjudicated events.

**Methods:**

TIMI adjudicated in a blinded fashion all potential hospitalization for heart failure (HHF) events, all potential MACE+ events previously adjudicated as not an endpoint event, and a random subset (~10%) of MACE+ events previously adjudicated as an endpoint event. An updated study‐level meta‐analysis of four randomized, placebo‐controlled, CV outcomes trials with dipeptidyl peptidase 4 (DPP‐4) inhibitors was then performed.

**Results:**

After re‐adjudication of potential HHF events in the intent‐to‐treat population, there were 224 patients with a confirmed event in the sitagliptin arm (1.05/100 person‐years) and 239 patients in the placebo arm (1.13/100 person‐years), corresponding to a hazard ratio (HR) of 0.94 (95% confidence interval [95% CI]: 0.78–1.13, *p* = .49). Concordance between the outcome of the original adjudication and the re‐adjudication for HHF events was 82.7%. The meta‐analysis of CV outcomes trials with DPP‐4 inhibitors with placebo and involving 43 522 patients yielded an HR of 1.07 (95% CI: 0.83–1.39), with moderate heterogeneity (*p* = .45, *I* ^2^ = 62.07%).

**Conclusion:**

The results of this independent re‐adjudication process and analyses of CV outcomes from TECOS were consistent with the original adjudication results and overall study findings. An updated study‐level meta‐analysis showed no overall significant risk for HHF with DPP‐4 inhibitors, but with statistical heterogeneity.

## 1 BACKGROUND

Sitagliptin, a dipeptidyl peptidase‐4 (DPP‐4) inhibitor, inhibits the metabolism and inactivation of the incretin hormones glucagon‐like peptide‐1 (GLP‐1) and glucose‐dependent insulinotropic polypeptide (GIP), and is approved as an adjunct to diet and exercise to improve glycemic control in adults with type 2 diabetes mellitus. The Trial Evaluating Cardiovascular Outcomes with Sitagliptin (TECOS) was a post‐approval, randomized, placebo‐controlled, double‐blind, event‐driven, multinational clinical trial conducted to assess the safety and efficacy of sitagliptin on cardiovascular (CV) outcomes.^1^, ^2^ The primary outcome, defined as the first confirmed event of the composite of CV death, nonfatal myocardial infarction, nonfatal stroke, or hospitalization for unstable angina (MACE+) in the per‐protocol (PP) population, occurred in 695 patients in the sitagliptin group (3.73 per 100 person‐years) and 695 patients in the placebo group (3.82 per 100 person‐years), with sitagliptin noninferior to placebo (hazard ratio [HR], 0.98; 95% confidence interval [95% CI], 0.88–1.09; *p* < .001). Risk of hospitalization for heart failure (HHF) did not differ between the groups (HR, 1.00; 95% CI, 0.83–1.20; *p* = .98) in the intent to treat (ITT) population.^3^ During TECOS, an independent clinical‐events classification committee led by the Duke Clinical Research Institute (DCRI), whose members were blinded to treatment assignment, adjudicated all potential CV events including death, myocardial infarction, stroke, hospitalization for unstable angina, and HHF before database lock.

Due to the association between some DPP‐4 inhibitors and heart failure (HF),^4^, ^5^, ^6^ all members in this drug class marketed in the United States currently carry warning language regarding HF. Supplemental New Drug Applications (sNDAs) for sitagliptin products were submitted to the US FDA to support addition of the results of TECOS to the products' prescribing information. Based on feedback from the FDA, the trial's Sponsor, Merck & Co., Inc., Kenilworth, NJ, USA, engaged a separate academic research organization also highly experienced in CV event adjudication, the TIMI Study Group (TIMI), to re‐adjudicate all potential HHF events, all potential MACE+ events previously adjudicated as not an endpoint event, and a random (approximately 10%) sample of MACE+ events previously adjudicated as an endpoint event.

In this paper, we describe the results of re‐adjudication on the CV outcomes of TECOS, as requested by FDA, and provide a study‐level meta‐analysis of four published, placebo‐controlled, CV outcomes trials with DPP‐4 inhibitors, updated with the re‐adjudication results from TECOS.

## 2 METHODS

**Figure 1:**
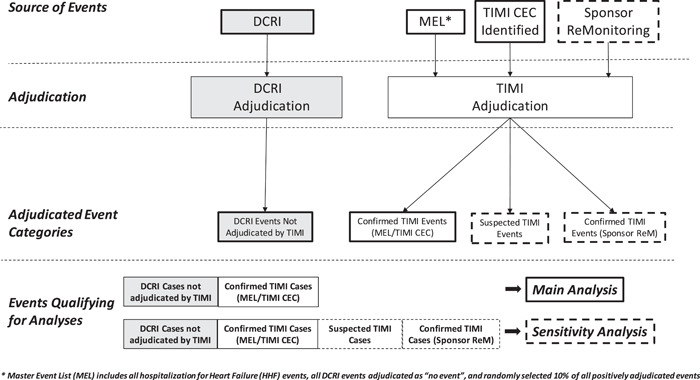
Event provenance of the clinical events in relation to main analysis (solid lines) and sensitivity analyses (dotted lines). CEC, Clinical Events Committee; DCRI, Duke Clinical Research Institute; MEL, Master Event List.

The methods and results of TECOS have been reported previously.^1^, ^2^ For the re‐adjudication project, events for the main and supplementary analyses were identified via three sources (Figure 1). For the main analyses, two sources were used. The first source was a Sponsor‐generated listing (Master Event List [MEL]) of events previously identified by the original DCRI triggering program for potential endpoint events. Of these events, 100% of previously adjudicated HHF events, 100% of previously negatively adjudicated (i.e., not confirmed as an endpoint event) MACE+ events, and approximately 10% of randomly selected, previously positively adjudicated (i.e., confirmed as an endpoint event) MACE+ events were re‐adjudicated by the TIMI Clinical Events Committee (CEC). The second source was via identification of previously unreported potential endpoint events by TIMI CEC Adjudicators or Medical Reviewers during their review of a submitted event. TIMI was able to query sites for additional supporting source documents. To support the main analyses, sensitivity analyses used a third source of previously unreported potential endpoint events that were submitted to TIMI as part of a Sponsor re‐monitoring program of select study sites that participated in TECOS. All events adjudicated by TIMI during this re‐adjudication project are referred to as “TIMI‐adjudicated” events. Both the original TECOS study and the re‐adjudication process were approved by the relevant ethics committees. All enrolled subjects in the TECOS study provided written informed consent.

The re‐adjudication project and database review of events conducted by TIMI were performed in a blinded manner without knowledge of treatment assignment or previous adjudication outcome of an event. TIMI adjudicated events based on the Clinical Events Classification Committee Charter for TECOS dated January 23, 2014,^1^ with the following exceptions: (1) suspected event—used in instances where the available documentation supported a diagnosis of MI, stroke, or HHF, but was insufficient to meet the charter definition; and (2) urgent HF visit—an HF event that meets all of the following: the patient has an urgent, unscheduled office/practice or emergency department visit for a primary diagnosis of HF, but not meeting the criteria for an HHF event, the patient exhibits new or worsening symptoms of HF on presentation, the patient has objective evidence of new or worsening HF, the patient receives initiation or intensification of treatment specifically for HF, with the exception that changes to oral diuretic therapy do not qualify as initiation or intensification of treatment.^7^ The TECOS trial utilized a combination of a manual and algorithm‐driven adjudication process for HF events which are summarized in Supporting Information: Table S1. For the TIMI adjudications, each event is assigned to two CEC Adjudicators who independently review the event and enter his or her adjudication. If the Adjudicators agree, then the event is complete and final. If the adjudication result is discordant, then the same CEC Adjudicator pair meet until consensus is reached. If consensus cannot be reached, the case is forwarded to the CEC Chair for final adjudication.

The categorization of the provenance of events identified in the re‐adjudication process, the flow of events through the adjudication process, and which events were included in the main and sensitivity analyses are summarized in Figure 1. For HHF events, MACE+ events previously negatively adjudicated, and the approximately 10% random subset of positively adjudicated MACE+ events, the main analyses of the re‐adjudication project included all confirmed events adjudicated by TIMI from the MEL and identified by the TIMI CEC and the remaining MACE+ events that were previously positively adjudicated (i.e., those not contained within the approximately 10% random sample) were not re‐adjudicated by TIMI. For all cases adjudicated by both TIMI and DCRI, the TIMI adjudication outcome was used in analyses. Sensitivity analyses included suspected events urgent HF for hospitalization events and cases identified during the Sponsor's re‐monitoring program.

Concordance, defined as the agreement between TIMI and DCRI (i.e., the proportion of the total number of events in agreement with respect to adjudication outcome among the total number of events adjudicated by both TIMI and DCRI), was calculated for HHF for all cases that were adjudicated by both TIMI and DCRI, as this is the only endpoint where every potential DCRI case was re‐adjudicated by TIMI. We also report the percent of events downgraded and upgraded, with DCRI adjudication being the base case.

Similar to the original TECOS Statistical Analysis Plan (SAP),^1^ the TIMI re‐adjudication project prespecified that the following hypotheses be tested in a sequential manner that began with testing for noninferiority for the primary composite CV outcome (MACE+) and key secondary composite CV outcome (MACE) in the PP population, with supporting analyses performed in the ITT population. Other secondary outcomes including HHF were evaluated for superiority in the ITT population. A total event analysis to account for multiple HHF events was performed under a counting process assumption based on the method described by Anderson‐Gill with robust variance estimator.

A study‐level meta‐analyses of four placebo‐controlled, CV outcomes trials of DPP‐4 inhibitors using random‐effects models on trial‐level data were performed for HHF and the composite of HHF or CV death from the following trials: SAVOR‐TIMI 53,^4^, ^5^ EXAMINE,^6^, ^8^ CARMELINA,^9^, ^10^ and TECOS using updated results based on re‐adjudication. Supporting Information: Table S2 summarizes the similar HHF definitions across studies. The HRs from each trial were combined by random effects model with a restricted maximum likelihood approach and Hartung‐Knapp adjustment to yield a point estimate of the pooled effect and 95% CI. The pooled effect describes a weighted average of the DPP‐4 inhibitor treatment effect on the outcomes of interest listed above, giving more weight to a trial with smaller variance as well as accounting for in‐between trial variability. Statistical heterogeneity across the two trials was assessed by Cochran's Q statistic and Inconsistency index (*I* ^2^)^11^. The meta‐analyses were conducted using R version 3.6.1 (R Core Team) and the R package metaphor.^12^ All analyses were performed by TIMI who had access to the raw data files from the original TECOS trial as well as the updated re‐adjudication results.

## 3 RESULTS

### 3.1 Re‐adjudication

A total of 14 671 patients underwent randomization in TECOS between December 2008 and July 2012 and were included in the ITT population. Median follow‐up was 3.0 years. Supporting Information: Table S3 presents the provenance of the 5568 individual events selected for re‐adjudication. The final re‐adjudication analysis included 2310 positively adjudicated MACE+ events, of which 620 (26.8%) were based on TIMI re‐adjudicated results and 1690 (73.2%) on the original DCRI adjudication results. By design, all of the 661 positively adjudicated HHF events included in the re‐adjudication analysis were based on TIMI adjudications. The concordance between the set of HHF endpoint events adjudicated by both TIMI and DCRI was 82.7% (825/998 cases). The re‐adjudication process downgraded 14.7% and upgraded 23.2% (Table 1).

**Table 1:** Concordance of TIMI and DCRI adjudication results for HHF

DCRI	TIMI			TIMI versus DCRI	
	HHF	Not a heart failure event	Total	Downgrade versus DCRI	Upgrade versus DCRI
HHF	590 (59.1%)	102 (10.2%)	692 (69.3%)	102 (14.7%)	‐
Not HHF	71 (7.1%)	235 (23.5%)	306 (30.7%)	‐	71 (23.2%)
Total	661 (66.2%)	337 (33.8%)	998 (100.0%)	‐	‐

Abbreviations: DCRI, Duke Clinical Research Institute; HHF, hospitalization for heart failure.

In both the PP and ITT populations, the results of the comparison between sitagliptin and placebo were nearly identical to the original TECOS findings (Table 2). Specifically, for the primary objective of the trial (to demonstrate noninferiority of sitagliptin vs. placebo) in the PP population using the re‐adjudication database, MACE+ occurred in 769 patients in the sitagliptin arm (10.6% overall event‐rate, 4.14 per 100 person‐years) and 760 patients in the placebo arm (10.5%, 4.20 per 100 person‐years), corresponding to an HR of 0.99 (95% CI: 0.90, 1.10, *p* for noninferiority <.0001). The corresponding HR for the original TECOS analysis was 0.98 (95% CI: 0.88, 1.09, *p* for noninferiority <.001). Likewise, the HR for the narrower MACE composite outcome in the PP population using the re‐adjudication database was 1.01 (95% CI: 0.91, 1.13, *p* for noninferiority <.001). The HR and 95% CI were virtually identical in the ITT population (Table 2). Sensitivity analyses that included suspected events and/or confirmed events via the Sponsor's remonitoring program provided similar results (Supporting Information: Table S4).

**Table 2:** TECOS primary analysis results after re‐adjudication

Endpoints	Per protocol						Intention to treat					
	Sitagliptin (*N* = 7257)		Placebo (*N* = 7266)				Sitagliptin (*N* = 7332)		Placebo (*N* = 7339)			
	*n* (%)	IR	*n* (%)	IR	HR (95% CI)^a^	*p*‐value^a^	*n* (%)	IR	*n* (%)	IR	HR (95% CI)^a^	*p*‐value^a^
Primary composite: CV death, nonfatal myocardial infarction, nonfatal stroke, or unstable angina requiring hospitalization (MACE+)	769 (10.6%)	4.14	760 (10.5%)	4.20	0.99 (0.90, 1.10)	<.0001	921 (12.6%)	4.48	924 (12.6%)	4.55	0.99 (0.90, 1.08)	.7966
Secondary composite: CV death, nonfatal myocardial infarction, or nonfatal stroke (MACE)	678 (9.3%)	3.62	655 (9.0%)	3.58	1.01 (0.91, 1.13)	<.0001	821 (11.2%)	3.96	805 (11.0%)	3.92	1.01 (0.92, 1.11)	.8252
Secondary outcome												
CV death	256 (3.5%)	1.33	238 (3.3%)	1.26	1.05 (0.88, 1.26)	.5668	388 (5.3%)	1.76	369 (5.0%)	1.68	1.04 (0.90, 1.20)	.5834
Fatal or non fatal myocardial infarction	335 (4.6%)	1.77	322 (4.4%)	1.74	1.02 (0.88, 1.19)	.7901	376 (5.1%)	1.79	377 (5.1%)	1.81	0.99 (0.86, 1.15)	.9277
Fatal or non fatal stroke	165 (2.3%)	0.86	184 (2.5%)	0.98	0.88 (0.71, 1.09)	.2383	186 (2.5%)	0.87	201 (2.7%)	0.95	0.92 (0.75, 1.12)	.4212
All cause death	343 (4.7%)	1.78	315 (4.3%)	1.67	1.07 (0.91, 1.24)	0.4136	548 (7.5%)	2.48	537 (7.3%)	2.45	1.01 (0.90, 1.14)	.8513
HHF^b^	202 (2.8%)	1.06	207 (2.8%)	1.11	0.97 (0.80, 1.17)	.7402	224 (3.1%)	1.05	239 (3.3%)	1.13	0.94 (0.78, 1.13)	.4901
CV death or HHF^b^	421 (5.8%)	2.21	410 (5.6%)	2.20	1.01 (0.89, 1.16)	.8393	540 (7.4%)	2.54	533 (7.3%)	2.54	1.01 (0.89, 1.14)	.8986

*Note*: IR is defined as the number of patients with events per 100 person‐years.

Abbreviations: CV, cardiovascular; HHF, hospitalization for heart failure; HR, hazard ratio; IR, incidence rate; TECOS, Trial Evaluating Cardiovascular Outcomes with Sitagliptin.

^a^ Based on the Wald statistic from a Cox PH model, stratified by region. For the composite endpoints, the *p‐*value corresponds to a test of noninferiority of sitagliptin, as compared with placebo, using a noninferiority margin of 1.3. For all other endpoints, the *p*‐value corresponds to a test of superiority.

^b^ Analyses of HHF were adjusted for a history of heart failure at baseline.

For HHF, after re‐adjudication, there were 224 patients with an event in the sitagliptin arm (1.05 per 100 person‐years) and 239 patients in placebo arm (1.13 per 100 person‐years) in the ITT population, corresponding to an HR of 0.94 (95% CI: 0.78, 1.13, *p* = .49). The HR for HHF in the same populations as the original TECOS analysis was 1.00 (95% CI: 0.83, 1.20, *p* = .98). In the total event analysis, there were 334 HHF events in patients assigned to sitagliptin and 359 events in patients assigned to placebo (HR: 0.93, 95% CI: 0.74–1.17, *p* = .54). As with MACE+ and MACE, sensitivity analyses for HHF that included suspected events and/or confirmed events via the Sponsor's remonitoring program provided similar results (Supporting Information: Table S4).

Urgent HF events were infrequent and balanced between treatment groups: seven patients in the sitagliptin arm, 0.1% overall event rate, 0.03 per 100 person‐years, and eight patients in placebo arm, 0.1%, 0.04 per 100 person‐years, corresponding to an HR of 0.88, 95% CI (0.32, 2.43).

### 3.2 Study‐level meta‐analysis of DPP‐4 studies

**Figure 2:**
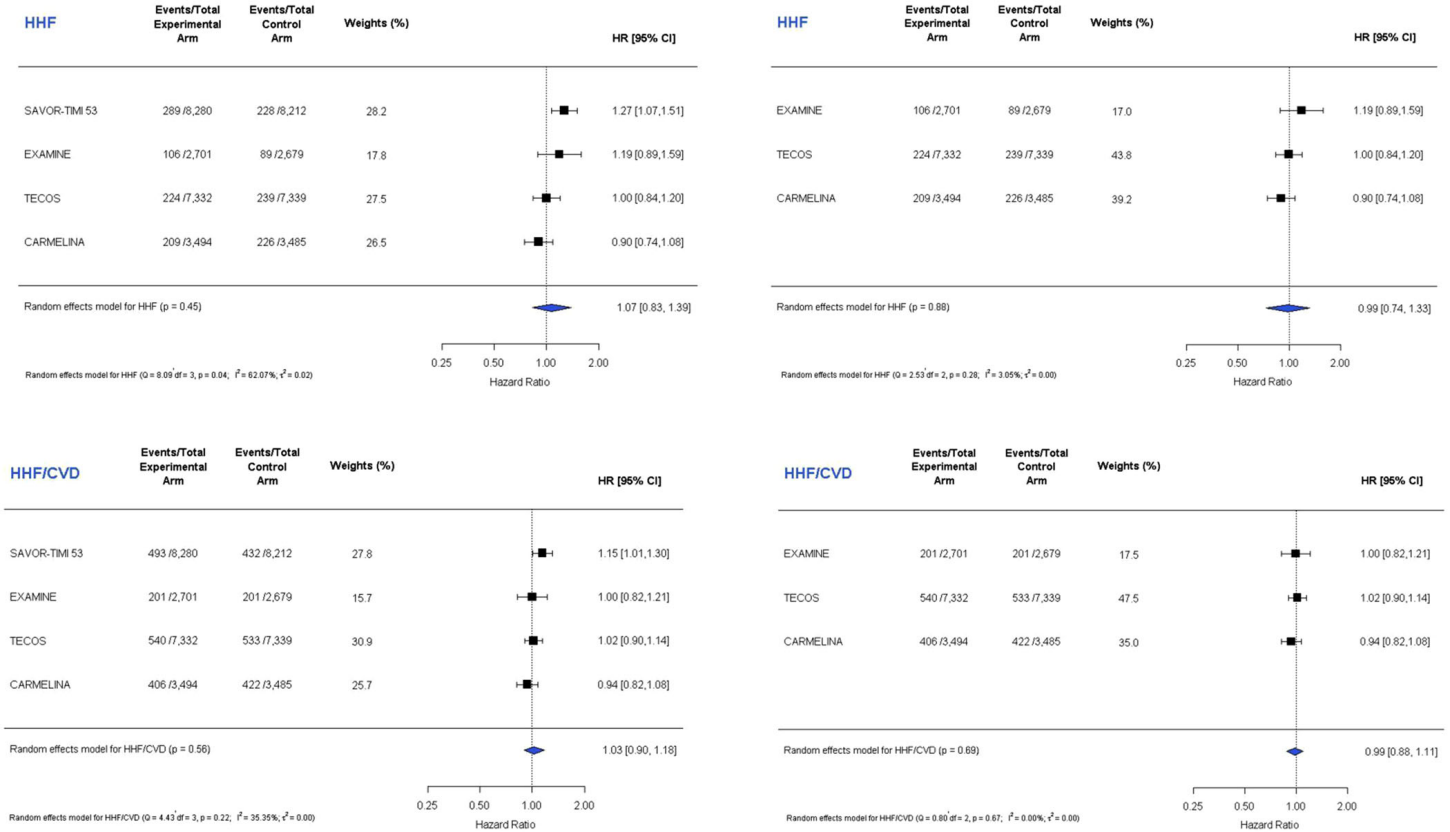
Study‐level meta‐analysis of the risk for HHF (top) and the composite of HHF or cardiovascular death (bottom) from the published outcomes trials of dipeptidyl peptidase‐4 (DPP‐4) inhibitors including the updated TECOS results from re‐adjudication. The left panels include all four studies. The right panels exclude SAVOR‐TIMI 53. HHF, hospitalization for heart failure; TECOS, Trial Evaluating Cardiovascular Outcomes with Sitagliptin.

**Figure 3:**
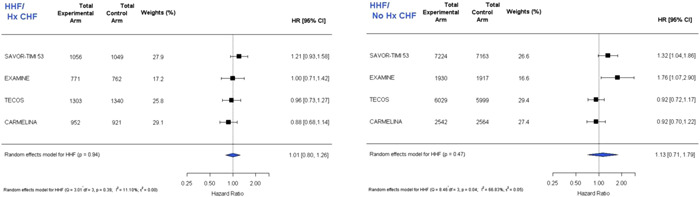
Study‐level meta‐analysis of the risk for HHF in patients with (left) and without (right) a history of heart failure before hospitalization from the published outcomes trials of dipeptidyl peptidase‐4 (DPP‐4) inhibitors including the updated TECOS results from re‐adjudication. HHF, hospitalization for heart failure; TECOS, Trial Evaluating Cardiovascular Outcomes with Sitagliptin.

A meta‐analysis of four published CV outcomes trials of DPP‐4 inhibitors versus placebo that includes the updated TECOS HHF results are presented in Figure 2. In total, there were 43 522 patients of whom 1610 had HHF. The pooled estimate for the effect of a DPP‐4 inhibitor on HHF was a HR of 1.07 (95% CI, 0.83–1.39), with moderate heterogeneity (*p* = .45, *I* ^2^ = 62.07%). The pooled estimate for the HHF or CV death composite was a HR of 1.03 (95% CI, 0.90–1.18), with mild heterogeneity (*p* = .56, *I* ^2^ = 35.35%). When SAVOR‐TIMI 53 was removed from the meta‐analysis, the corresponding numbers for HHF and HHF or CV death were HR: 0.99 (95% CI: 0.74, 1.33, *p* = .88, *I* ^2^ = 3.05%) and HR: 0.99 (95% CI: 0.88, 1.11, *p* = .69, *I* ^2^ = 0.00%), respectively, indicating no heterogeneity. Figure 3 presents a meta‐analysis for HHF stratifying by whether patients had a history of HF before randomization (*n* = 8154, HR: 1.01, 95% CI: 0.80–1.26, *p* = .94, *I* ^2^ = 11.10%) or no history of prior HF (*n* = 35 368, HR: 1.13, 95% CI: 0.71–1.79, *p* = .47, *I* ^2^ = 66.83%).

## 4 DISCUSSION

The results of this independent re‐adjudication process and analyses of TECOS were entirely consistent with the original adjudication results and study findings presented by the TECOS investigators in 2015. Specifically, the HRs and confidence intervals were nearly identical for the main analyses, supporting the noninferiority of sitagliptin compared to placebo for MACE+ and MACE. In addition, after systematically reviewing all potential HF events identified during the trial and in the re‐adjudication process, there was no difference between sitagliptin and placebo with respect to risk of first episode of HHF, the total number of HHF events, or the composite of CV death or HHF.

Using the same endpoint definition for HHF, the degree of concordance between the outcomes of the TIMI and the previous adjudications was high at 82.7%. We believe the small amount of discordance is to be expected considering these events were adjudicated more than 5 years apart, and in many cases with the benefit of additional clinical data being available to the TIMI CEC. Any discordance between the adjudication processes in determining which cases did or did not meet endpoint definitions, or through the identification of previously unreported events during this process, would not be expected to introduce any bias into the treatment effect of sitagliptin versus placebo as both adjudication processes were blinded to treatment assignment. TIMI, which did not participate in the original adjudication process, did not receive any unblinded data or treatment code until all adjudications were completed. The observed consistency of the sitagliptin and placebo comparisons between the two CEC results are therefore supportive of the hypothesis that there was no systemic bias in event identification or adjudication.

While this high level of concordance was consistent with our internal experience from other projects that compared the adjudications from two different CECs, this is one of the few examples of large‐scale systematic re‐adjudication of a clinical outcomes trial. In the re‐adjudication of the Rosiglitazone evaluated for CV outcomes in oral agent combination therapy for type 2 diabetes (RECORD) trial, re‐adjudication led to a disagreement in 21% of cases.^13^ There is no method to determine which adjudication in the discordant cases is “correct.” The high rate of concordance between two experienced academic research organizations is reassuring while the discordant cases highlight an inevitable variability in how even similar standardized definitions can be interpreted differently, in particular as the original adjudication process used a combined manual and algorithmic method while the re‐adjudication was entirely based on manual review. This is expected as source documents may not always be complete and the clinical events themselves are often complicated without a clearly identifiable primary diagnosis. Moreover, even with the same definitions, different CEC can develop different conventions for handling challenging cases Randomization and blinded adjudication prevent bias.

We prospectively defined urgent HF visits as a sensitivity analysis in cases for which the stricter definitions of HHF may have missed less severe, but clinically relevant, events. There were few urgent HF visits events identified in this project, which may be due to low utilization of this management strategy when TECOS was conducted or because investigators were not specifically instructed to report urgent HF events in TECOS and therefore those cases never reached the DCRI CEC during conduct of the trial.

The results of the updated study‐level meta‐analysis demonstrate a consistent lack of effect of DPP‐4 inhibitors on HHF, apart from saxagliptin. Whether the observation of an increased risk of HHF with saxagliptin was due to an unexpected “off‐target” effect of saxagliptin^14^ or due to chance is not known. Saxagliptin, for example, did not increase levels of natriuretic peptides or high‐sensitivity troponin level compared to placebo.^5^ The Mechanistic Evaluation of Glucose‐lowering Strategies in Patients With Heart Failure (MEASURE‐HF) (ClinicalTrials.gov Identifier: NCT02917031) found no difference in left ventricular dimensions, function, or levels of natriuretic peptides between saxagliptin, sitagliptin, or placebo in 348 patients with HF and reduced ejection function. {Pitt, 2021 #910} When the SAVOR‐TIMI 53 trial was removed from the pooled analysis, the HR for HHF or the composite of CV death or HHF was 0.99 with no heterogeneity, which is reassuring for the remaining members of the class. The cumulative data from the randomized trials may support the removal of cautionary language regarding HF from some of the DPP‐4 FDA labeling. These results are in contrast to several observational studies that conversely found that DPP‐4 inhibitor use (including saxagliptin) was associated with lower rates of HHF.^15^, ^16^, ^17^, ^18^ Recently, the FDA Sentinel program propensity‐matched >350 000 patients on sitagliptin and saxagliptin and found numerically or statistically significantly lower rates of HHF with these drugs versus other glucose‐lowering agents and a lower rate with saxagliptin than with sitagliptin.^19^ These findings from uncontrolled observational data run counter to the observations in carefully conducted randomized, placebo‐controlled trials, raising the concern that the residual confounding or bias in the observational studies related prescribing patterns or patient risk could not be completely addressed, even with rigorous statistical methodology.

In summary, after a prospective, comprehensive, and systematic re‐adjudication of all HHF events in TECOS, we confirmed that there was no difference between sitagliptin and placebo with respect to the risk of HHF.

## Supporting information

Supporting information.

## Data Availability

The trial database cannot be shared, but parties interested in collaborating should contact the corresponding author.
