# Supplementary material for: Re‐adjudication of the Trial Evaluating Cardiovascular Outcomes with Sitagliptin (TECOS) with study‐level meta‐analysis of hospitalization for heart failure from cardiovascular outcomes trials with dipeptidyl peptidase‐4 (DPP‐4) inhibitors
Source: Clin Cardiol. 2022 Jun 17;45(7):794–801. doi: 10.1002/clc.23844 (PMC9286326; doi:10.1002/clc.23844)
Supplement: Supplementary file 1 — Supporting information. [file CLC-45-794-s001.docx]

Scirica et al

Supplemental Table 1

TECOS Heart Failure Adjudication Process

Congestive Heart Failure (CHF) events will be reviewed using the following approach: (Please note that validation of the algorithm will be done by a clinician (CECC coordinator or CECC MD)

1. Data will be collected on the eCRF based on the endpoint definition
2. A Computer Algorithm will be created to assess the data on the eCRF
3. The algorithm will be a series of logic statements evaluating the eCRF data to determine if the data provided is consistent and supports the endpoint criteria.
4. If the data are consistent and data are complete on the eCRF then the algorithm will determine if an endpoint CHF event did or did not occur. If the data are non consistent or data are not complete despite query process, then the algorithm will not make a determination and the event will be reviewed by one CECC MD and a determination made about whether an endpoint CHF event did or did not occur.
5. The algorithm will be validated by a clinician. The clinician will review the algorithmic determinations about whether a CHF event did or did not occur for those that a decision is made for the first 25 events.
6. If there are discrepancies found in the algorithm the CECC coordinator will note these to the data manager and have all CHF events reviewed manually until the discrepancy is resolved.
7. A clinician will validate the algorithm on the next 10 CHF events after the discrepancy has been resolved to ensure that it supports the endpoint criteria.

Supplemental Table 2

**Definitions for Hospital for Heart Failure Definitions**

|  | **SAVOR-TIMI 53** | **EXAMINE** | **TECOS** | **CARMELINA** |
| --- | --- | --- | --- | --- |
| **Hospitalization** | An admission to an inpatient unit or a visit to an emergency department that results in at least a 12 hour stay (or a date change if the time of admission/discharge is not available). | An inpatient admission or an emergency department visit of more than 12 h | Admission to an inpatient unit or a visit to an emergency department that result in at least a 12 hour stay (or a date change if the time of admission/discharge is not available). | Inpatient admission or a ≥12-hour stay in the emergency department as a result of clinical manifestations of new or worsening HF. |
|  | ***AND*** | | | |
| **Clinical Manifestation** | New or worsening dyspnea, orthopnea, paroxysmal nocturnal dyspnea, edema, pulmonary basilar crackles, jugular venous distension, new or worsening third heart sound or gallop rhythm, or radiological evidence of worsening heart failure. | New or worsening dyspnoea, orthopnoea, paroxysmal nocturnal dyspnoea, peripheral oedema, bibasilar rales on pulmonary examination, jugular venous distention, new third heart sound, radiographic evidence of heart failure | New or worsening: dyspnea, orthopnea, paroxysmal nocturnal dyspnoea, edema, pulmonary basilar crackles, jugular venous distension, or radiological evidence of worsening heart failure. | Dyspnea, orthopnea, paroxysmal nocturnal dyspnea, edema, rales, jugular venous distension, third heart sound or gallop rhythm, and/or radiological evidence of HF. |
|  | ***AND*** | | | |
| **Increased Therapy** | Additional/Increased therapy: Initiation of intravenous diuretic, inotrope, or vasodilator therapy; Uptitration of intravenous therapy, if already on therapy; Initiation of mechanical or surgical intervention (mechanical circulatory support, heart transplantation or ventricular pacing to improve cardiac function), or the use of ultrafiltration, hemofiltration, or dialysis that is specifically directed at treatment of heart failure. | Parenteral diuretic, inotropic, or vasodilator therapy, ultrafiltration or dialysis, or mechanical or surgical intervention (including heart transplant | Additional / increased therapy:  a. Intravenous treatment with diuretic, inotrope, or vasodilator therapy  OR  b. Mechanical or surgical intervention (mechanical circulatory support, heart  transplantation or ventricular pacing to improve cardiac function,) or the  use of ultrafiltration, hemofiltration or dialysis that is specifically directed  at treatment of heart failure. | Added or increased  therapy that included (1) initiation or up-titration of diuretics, inotropes, and/or vasodilator therapy or (2) initiation of mechanical or surgical therapy, such as mechanical circulatory support, heart transplantation, or ventricular pacing  to improve cardiac function, and/or (3) use of ultrafiltration, hemofiltration, or dialysis directed at the treatment of HF. |

**Supplemental Table 3**

Provenance of Events included in TIMI Re-adjudication Process

|  | ***DCRI-Adjudicated Events*** | ***TIMI-adjudicated Events*** | | | ***Duplicate Events**** |  |
| --- | --- | --- | --- | --- | --- | --- |
| ***Endpoint Categories*** | ***n*** | ***MEL*** | ***TIMI CEC IDENTIFIED*** | ***SPONSOR MONITOR IDENTIFIED*** |  | ***TOTAL*** |
| DEATH | 649 | 435 | 1 | 5 | 0 | 1090 |
| Cardiac Ischemic Events | 803 | 1771 | 48 | 74 | 35 | 2696 |
| Cerebrovascular Events | 327 | 335 | 15 | 8 | 1 | 685 |
| HOSPITALIZATION FOR HEART FAILURE | 0 | 1001 | 86 | 10 | 8 | 1097 |
| Total | 1779 | 3542 | 150 | 97 | 44 | 5568 |

* Duplicate Events occurred when a case was identified via different sources. These cases were not included in the final analysis.

Master Events Listing (MEL) includes all or partial events selected for re-adjudication for TIMI and previously adjudicated by DCRI.

Supplemental Table 4: TECOS Sensitivity Analyses that included Sponsor Monitor identified events and suspected events (Sensitivity Analysis)

|  |  |  | ***Per Protocol*** |  |  |  |  |  | ***Intention to Treat*** |  |  |  |
| --- | --- | --- | --- | --- | --- | --- | --- | --- | --- | --- | --- | --- |
|  | ***Sitagliptin (N=7257)*** |  | ***Placebo (N=7266)*** |  |  |  | ***Sitagliptin (N=7332)*** |  | ***Placebo (N=7339)*** |  |  |  |
| ***Endpoints*** | ***n (%)*** | ***IR*** | ***n (%)*** | ***IR*** | ***HR (95% CI) †*** | ***P value †*** | ***n (%)*** | ***IR*** | ***n (%)*** | ***IR*** | ***HR (95% CI) †*** | ***P value †*** |
| **PRIMARY COMPOSITE: CARDIOVASCULAR DEATH, NONFATAL MYOCARDIAL INFARCTION, NONFATAL STROKE, OR UNSTABLE ANGINA REQUIRING HOSPITALIZATION (MACE+)** | 782 (10.8%) | 4.22 | 783 (10.8%) | 4.33 | 0.98 (0.89 ,1.08) | <0.0001 | 938 (12.8%) | 4.57 | 947 (12.9%) | 4.67 | 0.98 (0.90 ,1.07) | 0.6747 |
| **SECONDARY COMPOSITE: CARDIOVASCULAR DEATH, NONFATAL MYOCARDIAL INFARCTION, OR NONFATAL STROKE (MACE)** | 690 (9.5%) | 3.69 | 676 (9.3%) | 3.70 | 1.00 (0.90 ,1.11) | <0.0001 | 837 (11.4%) | 4.04 | 825 (11.2%) | 4.02 | 1.00 (0.91 ,1.11) | 0.9212 |
| **SECONDARY OUTCOME** |  |  |  |  |  |  |  |  |  |  |  |  |
| CARDIOVASCULAR DEATH | 256 (3.5%) | 1.33 | 238 (3.3%) | 1.26 | 1.05 (0.88 ,1.26) | 0.5668 | 390 (5.3%) | 1.77 | 370 (5.0%) | 1.69 | 1.04 (0.90 ,1.20) | 0.5609 |
| FATAL OR NON FATAL MYOCARDIAL INFARCTION | 350 (4.8%) | 1.85 | 343 (4.7%) | 1.86 | 1.00 (0.86 ,1.16) | 1.0000 | 139 (1.9%) | 0.65 | 153 (2.1%) | 0.72 | 0.91 (0.72 ,1.15) | 0.4250 |
| FATAL OR NON FATAL STROKE | 171 (2.4%) | 0.89 | 197 (2.7%) | 1.05 | 0.85 (0.69 ,1.05) | 0.1280 | 393 (5.4%) | 1.87 | 400 (5.5%) | 1.92 | 0.98 (0.85 ,1.12) | 0.7437 |
| ALL CAUSE DEATH | 343 (4.7%) | 1.78 | 315 (4.3%) | 1.67 | 1.07 (0.91 ,1.24) | 0.4136 | 198 (2.7%) | 0.93 | 218 (3.0%) | 1.03 | 0.90 (0.75 ,1.10) | 0.3009 |
| HOSPITALIZATION FOR HEART FAILURE § | 224 (3.1%) | 1.18 | 222 (3.1%) | 1.19 | 1.00 (0.83 ,1.21) | 0.9919 | 551 (7.5%) | 2.50 | 539 (7.3%) | 2.46 | 1.01 (0.90 ,1.14) | 0.8293 |
| CV DEATH OR HOSPITALIZATION FOR HEART FAILURE § | 439 (6.0%) | 2.30 | 423 (5.8%) | 2.27 | 1.03 (0.90 ,1.17) | 0.7072 | 247 (3.4%) | 1.16 | 258 (3.5%) | 1.23 | 0.96 (0.80 ,1.14) | 0.6285 |
|  |  |  |  |  |  |  | 556 (7.6%) | 2.62 | 548 (7.5%) | 2.61 | 1.01 (0.90 ,1.14) | 0.8674 |

IR (Incidence rate) is defined as the number of patients with events per 100 person years.

†Based on the Wald statistic from a Cox PH model, stratified by region. For the composite endpoints, the P value corresponds to a test of noninferiority of sitagliptin, as compared with placebo, using a noninferiority margin of 1.3. For all other endpoints, the p value corresponds to a test of superiority.

§ Analyses of hospitalization for heart failure were adjusted for a history of heart failure at baseline.
